# Detach GaN-Based Film to Realize a Monolithic Bifunctional Device for Both Lighting and Detection

**DOI:** 10.3390/nano13020359

**Published:** 2023-01-16

**Authors:** Pan Dai, Ziwei Xu, Min Zhou, Min Jiang, Yukun Zhao, Wenxian Yang, Shulong Lu

**Affiliations:** 1 School of Information Engineering, Huzhou University, Huzhou 313000, China; 2 Nano-Devices and Materials Division, Suzhou Institute of Nano-Tech and Nano-Bionics (SINANO), Chinese Academy of Sciences (CAS), Suzhou 215123, China; 3 School of Nano-Tech and Nano-Bionics, University of Science and Technology of China (USTC), Hefei 230026, China

**Keywords:** self-powered photodetector, lift-off (In,Ga)N film, monolithic bifunctional device, ultraviolet/visible reject ratio

## Abstract

Due to the emerging requirements of miniaturization and multifunctionality, monolithic devices with both functions of lighting and detection are essential for next-generation optoelectronic devices. In this work, based on freestanding (In,Ga)N films, we demonstrate a monolithic device with two functions of lighting and self-powered detection successfully. The freestanding (In,Ga)N film is detached from the epitaxial silicon (Si) substrate by a cost-effective and fast method of electrochemical etching. Due to the stress release and the lightening of the quantum-confined Stark effect (QCSE), the wavelength blueshift of electroluminescent (EL) peak is very small (<1 nm) when increasing the injection current, leading to quite stable EL spectra. On the other hand, the proposed monolithic bifunctional device can have a high ultraviolet/visible reject ratio (*Q* = 821) for self-powered detection, leading to the excellent detection selectivity. The main reason can be attributed to the removal of Si by the lift-off process, which can limit the response to visible light. This work paves an effective way to develop new monolithic multifunctional devices for both detection and display.

## 1. Introduction

Due to their advantages of low energy consumption, long lifetime, small volume, etc., light-emitting diodes (LEDs) based on (In,Ga)N materials have been extensively utilized in the solid-state lighting, display and communication fields [1,2,3]. By adjusting the Indium (In) component, the energy bandgap of (In,Ga)N can be modulated from 0.67 eV (InN) to 3.4 eV (GaN), which covers the whole visible range [4]. (In,Ga)N can also be an excellent candidate for fabricating photodetectors (PDs) because of its high stability and direct and tunable bandgap, which can be applied in the systems of medical, communication and environmental monitoring [5,6,7,8]. In the conventional way, the dual functionalities of light emission and detection can be realized by combining a LED and a PD, which makes the system more cumbersome and expensive [9,10,11]. Thus, with the increasing demand for the miniaturization, portability and multifunctionality of optoelectronic products, the ability to integrate LEDs and PDs within a single chip is essential for next-generation optoelectronic devices [10,12,13,14].

PDs operating at a zero-voltage bias can have self-powered characteristics [15], which are promising to be utilized as wireless and implantable devices in harsh working environments by alleviating the energy consumption [16,17]. Due to the outstanding advantages of low-cost, low-power consumption and simple fabrication processes, photoelectrochemical (PEC) PDs have attracted extensive attention for fabricating self-powered PDs [15,17,18]. Therefore, it has the potential to open up a new branch of industry by merging a LED and a self-powered PEC PD into a monolithic device.

According to the epitaxial substrates, silicon (Si) has received much attention due to its easy processing properties, low price and good electrical and thermal conductivity [19,20,21]. Indeed, the possibility of crack-free epitaxial GaN film growth on Si has been demonstrated by using various routes such as AlN, SiC and (Al,Ga)N buffer layers [22,23,24]. However, the Si substrate can produce an unnecessary light response to visible light, resulting in a low ultraviolet (UV)/visible reject ratio [16,20]. Although it is a key parameter for PDs, the UV/visible reject ratio is very difficult to be enhanced significantly due to a key reason of visible interference [16,25]. In our previous work, we demonstrated a novel electrochemical (EC) procedure for transferring the GaN-based nanowires from Si to foreign substrates [16,26,27,28]. However, such previous works have not realized the bifunctional (In,Ga)N device of both lighting and self-powered detection. Hence, it is still quite attractive but highly challenging to realize the monolithic device with both functions of lighting and self-powered detection.

In this work, we utilize the EC method to detach (In,Ga)N films from the original Si substrates to prepare the monolithic bifunctional device of lighting and self-powered detection. The photoresponse performance is studied at different incident light powers. Meanwhile, the luminescence performance of the same device is also characterized by the injecting current. The proposed bifunctional device has the potential significance for novel multifunctional integrated chips.

## 2. Experimental Methods

### 2.1. Preparation and Lift-Off Procedure of (In,Ga)N Film

**Figure 1:**
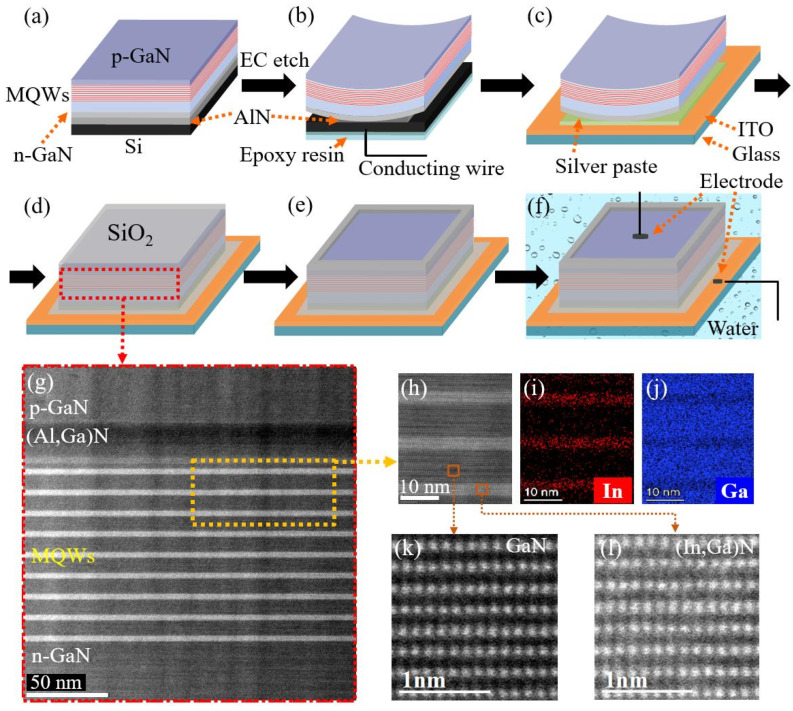
(**a**) Schematic diagram of the (In,Ga)N/GaN epitaxial structure on Si. (**b**) Detach the film by the EC method. (**c**) Transfer the flexible film onto the indium–tin–oxide (ITO) surface, connecting by silver paste. (**d**) Deposit the thick silica (SiO_2_) passivation layer. (**e**) Selectively etch the SiO_2_ layer. (**f**) Fabricate the metal electrodes on the GaN and ITO surfaces, then operate the device as a PEC PD under water. (**g**) Side-view STEM image of the GaN-based film after EC etching. (**h**) Enlarged scanning transmission electron microscope (STEM) image of the (In,Ga)N/GaN MQWs. High-resolution EDX mapping of the (**i**) In element and (**j**) Ga element within the (In,Ga)N/GaN MQWs. Aberration-corrected STEM (AC-STEM) images of (**k**) GaN and (**l**) (In,Ga)N crystals.

As shown in Figure 1a, GaN-based samples were epitaxially grown on Si(111) by metal organic chemical vapor deposition (MOCVD). Along the growth direction, the epitaxial structure consisted of a ~300-nm-thick AlN nucleation layer (*T_G_* (Growth temperature) ≈ 1080 °C), a ~450-nm-thick (Al,Ga)N multilayer buffer (*T_G_* ≈ 1050 °C), an ~800-nm-thick unintentionally doped (UID) GaN layer (*T_G_* ≈ 1020 °C), a ~2800-nm-thick Si-doped n-GaN ([Si] ≈ 8 × 10^18^ cm^−3^; *T_G_* ≈ 1020 °C), 9-period ~3-/10-nm-thick (In,Ga)N/GaN multiple quantum wells (MQWs, *T_G_* ≈ 750 °C), a ~30-nm-thick Mg-doped p-(Al,Ga)N electron-blocking layer (EBL, [Mg] ≈ 1 × 10^20^ cm^−3^; *T_G_* ≈ 940 °C), a ~60-nm-thick Mg-doped p-GaN layer ([Mg] ≈ 3 × 10^19^ cm^−3^; *T_G_* ≈ 940 °C) and a ~20-nm-thick heavily Mg-doped p-GaN contact layer ([Mg] ≈ 2 × 10^20^ cm^−3^; *T_G_* ≈ 940 °C). After the MOCVD growth, the as-grown samples were divided into small pieces. These pieces were then ultrasonically cleaned in acetone and isopropanol for 10 min, respectively. Next, the Sn/Pb alloys were melted as electrical contacts to connect the Si and a conductive wire by an electric soldering iron (Figure 1b). To avoid corrosion during the EC process, the surfaces of these electrical contacts were coated with epoxy resin. In the EC process, the sample and the Pt sheet immersed in a 1 mol/L nitric acid (HNO_3_) solution were functioned as the anode and cathode, respectively. More details can be found in Refs. [26,29].

### 2.2. Fabrication of Monolithic Device

When the (In,Ga)N film was detached from the Si substrate, it was retrieved and rinsed off with deionized water. Before the transfer process, the silver paste was smoothed on the top surface of the ITO layer by a squeegee (Figure 1c). Subsequently, the (In,Ga)N film was transferred to the surface of silver paste (Figure 1c,d). Then, the sample was kept at 130 °C for 30 min to solidify the silver paste. As shown in Figure 1d, a SiO_2_ passivation layer was deposited on the top and side surfaces of the film to prevent the current leakage and EC etching. After that, the SiO_2_ layer was selectively etched to expose the top surface of the GaN layer (Figure 1e). Finally, the metal electrodes were fabricated on the top surfaces of the GaN and ITO layers, respectively (Figure 1f). More details of calibrating *P_inc_* can be found in Ref. [16].

### 2.3. Characterization and Measurement Methods

Lighting measurements do not need electrolytes, while the measurements of PEC detection need them (e.g., pure water) for oxidation-reduction reactions (Figure 1f) [17,27]. The effective area of the device (*S_device_*) is ~0.06 cm^2^. The responsivity (*R*) is another key parameter of the PD, which is calculated by the following equation [30]:

(1) $$R=\frac{I_{ph}}{S_{device}\cdot \;P_{inc}},$$

where *P_inc_* is the incident light–power density. As a key optical property, the electroluminescence (EL) spectra can be achieved by injecting the current. Raman scattering experiments were carried out with a 532 nm laser, which were collected by a spectrometer (LABRAM HR). To characterize the morphology and element distribution, a STEM (Talos F200X, FEI) with a high-resolution energy dispersive X-ray (EDX) mapping and spherical AC-STEM was utilized. A focused ion beam (FIB, Scios, FEI) was utilized to prepare the STEM samples. An EC workstation with a PEC system (DH 7000) was used to evaluate the electrical properties of PDs in a reaction vessel with pure water at 0 V bias. The PD characteristics were all measured under the lighting sources of LEDs.

## 3. Results and Discussion

As shown in Figure 1g, clear (In,Ga)N/GaN and (Al,Ga)N/GaN interfaces can be achieved in the regions of (In,Ga)N/GaN MQWs, (Al,Ga)N EBL and p-GaN. It demonstrates that the epitaxial structure agrees well with the design. Figure 1h–j illustrate the regularly spaced (In,Ga)N/GaN interfaces without obvious dislocations in the MQW region in compositions that are uniformly distributed within the (In,Ga)N quantum well. Furthermore, the clear lattice fringes of both GaN and (In,Ga)N crystals can be observed in Figure 1k,l, indicating the good crystallinity of the active region for the device.

**Figure 2:**
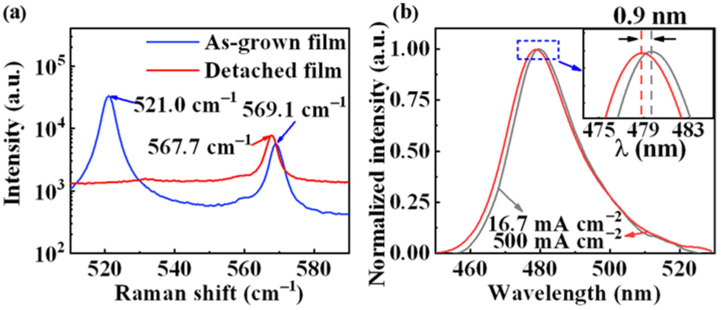
(**a**) Raman spectra of the detached and as-grown films. (**b**) EL spectra of the monolithic device based on the detached film when injecting the current.

To characterize the stress changes before and after detaching the films from the Si substrate, Raman scattering spectra were measured and shown in Figure 2a. For the as-grown sample before EC etching, two strong peaks exist around 569.1 cm^−1^ and 521.0 cm^−1^, corresponding to the wurtzite-structured GaN (E_2_ (high)) and Si–Si bond, respectively. After EC etching, the peak corresponding to the Si–Si bond disappears, resulting from the removal of Si. Furthermore, the E_2_ (high) peak for GaN is shifted to be 567.7 cm^−1^, which mainly results from the stress release within the films. Such a blue-shifted GaN peak of 1.4 cm^−1^ corresponds to a stress release of 2.61 GPa [29], which could reduce the internal polarization effects. As clearly illustrated in Figure 2b, the EL spectra demonstrate the lighting function of a monolithic device. When increasing the injection current from 16.7 to 500 mA/cm^2^, the cyan peak only shifts from 479.7 nm to 478.8 nm (<1 nm). In comparison, such a peak blueshift is normally larger than 2 nm for conventional LEDs without the lift-off process [31,32]. As a result, the peak wavelength of the monolithic device is quite stable at different current densities.

**Figure 3:**
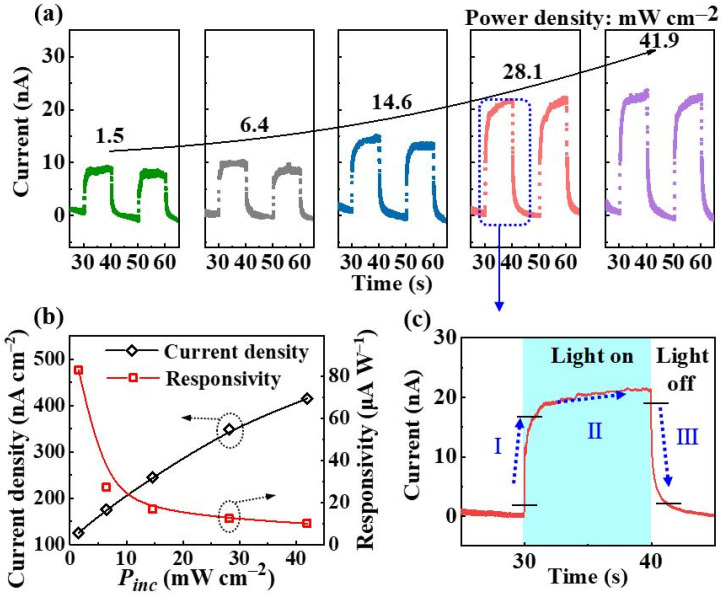
(**a**) Photo-switching behaviors of the self-powered PEC PD under 365 nm illumination with different incident light–power densities (Unit: mW cm^−2^). Different colors indicate different incident light–power densities. (**b**) Photocurrent density and responsivity as a function of the incident light–power density. (**c**) Response of PEC PD under 365 nm illumination at a zero-voltage bias.

Figure 3a illustrates the regular time-dependent photocurrent characteristics. As the detection measurements can be accomplished at 0 V bias [15,17], the monolithic device is demonstrated to have another function of self-powered detection. With the increase in the incident light–power intensity, the photocurrent (*I*_ph_) increases simultaneously (Figure 3a,b), exhibiting a characteristic of light–power dependence. At a low *P_inc_*, the *R* can reach 83 μA W^−1^ (Figure 3b). As the key parameters, rise time (*t_r_*) is defined as the time the photocurrent rises from 10% to 90% of the peak photocurrent, and decay time (*t_d_*) is defined as the time the photocurrent falls from 90% to 10% of the peak photocurrent [33]. As shown in Figure 3c, the rise time and decay time are 0.89 s and 1.26 s, respectively.

To evaluate the detection selectivity of a device before and after the lift-off process, the UV/visible reject ratio (*Q*) is generally used, which is calculated by the following equation:

(2) $$Q=\frac{R_{\lambda 1}}{R_{\lambda 2}}.$$

$R_{\lambda 1}$ and $R_{\lambda 2}$ represent the *R* data at λ1 (365 nm) and λ2 (620 nm) wavelengths, respectively. As clearly shown in Table 1, the *Q* data of the monolithic device based on the detached film can reach 821, which is high compared to those of the other PEC PDs.

**Figure 4:**
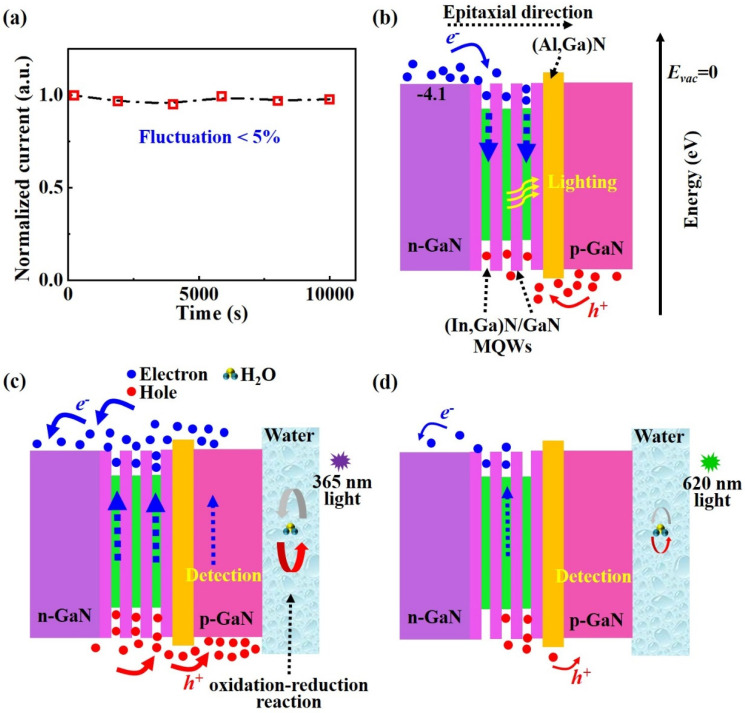
(**a**) Long-time photocurrent behavior of PD. (**b**) The energy band diagram of the monolithic device under the current injection (function of lighting). The energy band diagram of the device under (**c**) 365 nm illumination and (**d**) 620 nm illumination (function of detection). For a better illustration, the 9-period (In,Ga)N/GaN MQWs are simplified as 3-period MQWs.

In addition, with the PD continuously working from 0 to ~10,000 s, the photocurrent stays stable (Figure 4a). In general, GaN-based materials have excellent stability. Due to the negligible fluctuation (<5%), the essentially unchanged photocurrent indicates the outstanding stability of the monolithic device.

To better study the underlying mechanism of the monolithic bifunctional device, the schematic illustrations are plotted in Figure 4b–d, which show the energy band diagrams when operating in the lighting and detection states. As shown in Figure 4b, when forward bias is applied on both electrodes of the device (Figure 1f), electrons and holes are injected into the active region of MQWs in opposite directions under the action of the electric field. The piezoelectricity-induced quantum-confined Stark effect (QCSE) is the key factor contributing to the effect of the blueshift [31]. The detached film can release the strong strain, which partially weakens the QCSE and restores the energy band, mainly leading to a small blueshift (<1 nm, Figure 2b).

Under illumination, carriers are excited to the valence band (*E_V_*) and conduction band (*E_C_*) to produce electron–hole pairs (Figure 4c). Photogenerated holes transport to pure water, while the electrons transport to the n-GaN layer. Hence, the directions of carrier transport within the energy bands are opposite at both states of lighting and detection. When the GaN layer contacts the electrolyte of the water, an EC equilibrium is established by conveying excess carriers through the top and side film/water interfaces. An internal electric field (i.e., built-in field) formed in the (In,Ga)N/GaN heterojunction and appropriate energy level alignment can enhance the efficiency of carrier separation and transport [40,41]. Under 365 nm illumination, the photocurrent can be generated by the following reactions:

(3) $$4H^{+}+4e^{-}=2H_{2},$$

(4) $$4h^{+}+2H_{2}O=O_{2}+4H^{+}.$$

when the 365 nm light on (Process I in Figure 3c), the photocarriers can be immediately generated within the GaN and (In,Ga)N layers. Then, the photogenerated holes can easily move towards the film/water interfaces [42]. After that, the current density recovers to a new steady state under continuous illumination (Process II in Figure 3c). With the light off, the transport of existing carriers in the bands continues, which mainly results in the rapid decrease of the current (Process III in Figure 3c). Then, the current density recovers to a new steady state again.

In addition, all the GaN, (In,Ga)N and (Al,Ga)N layers cannot absorb 620 nm photons (2.0 eV) because of the large energy bandgaps (*E_g_* > 2.0 eV), while the Si substrate can due to the low energy bandgaps (*E_g_* < 2.0 eV). That means Si substrates are able to generate a certain photocurrent under 620 nm illumination. Thus, the removal of Si by the lift-off process can limit the response to visible light (Figure 4d), mainly leading to enhancing the UV/visible reject ratio (821, Table 1) to achieve excellent detection selectivity.

## 4. Conclusions

In this work, a monolithic bifunctional device was demonstrated successfully to have both functions of lighting and self-powered detection. This monolithic device is realized by a lift-off (In,Ga)N film, which is detached from the original Si substrate by EC etching. When increasing the injection current significantly, the blueshift of the peak EL wavelength is smaller than 1 nm, leading to very stable luminescent spectra. The main reason can be attributed to the release of stress and the weakening of piezoelectricity-induced QCSE. Moreover, for self-powered detection, the monolithic bifunctional device has a high UV/visible reject ratio. Thanks to the removal of the original Si substrate by the lift-off process, such an excellent characteristic of detection selectivity mainly results from the response limitations of the visible light. Therefore, the monolithic bifunctional device has broad application prospects in the fields of solid-state lighting, communication and environmental monitoring where low cost, portability and low-power consumption are required.

## Figures and Tables

**Table 1:** Comparison between this work and other recently reported PDs.

Material	Light Source (UV/Visible, nm)	PEC-Type PD	Applied Bias	UV/Visible Reject Ratio (*Q*)	Reference
As-grown (In,Ga)N film	365/620	Yes	Self-powered	587	This work
Detached (In,Ga)N film	365/620	Yes	Self-powered	821	This work
(Al,Ga)N nanowires	310/490	Yes	Self-powered	485	[16]
Pt/GaN	350/500	Yes	Self-powered	185	[25]
GaN/MoO_3-x_	355/400	No	Self-powered	20000	[34]
ZnO/CdS/p-GaN	300/500	Yes	Self-powered	48	[35]
(Al,Ga)N nanowires	310/490	Yes	Self-powered	977	[28]
α-Ga_2_O_3_	260/400	Yes	Self-powered	34	[36]
Pt/(Al,Ga)N	254/365	Yes	Self-powered	144	[17]
Ga_2_O_3_/Al_2_O_3_	264/400	Yes	Self-powered	35	[37]
In_2_O_3_ MR	365/455	Yes	0.6 V	21	[38]
ZnGa_2_O_4_ layers	240/470	No	5.0 V	84	[39]

## Data Availability

The data presented in this study are available upon request from the corresponding author.
